# Increased curative treatment is associated with decreased prostate cancer‐specific and overall mortality in senior adults with high‐risk prostate cancer; results from a national registry‐based cohort study

**DOI:** 10.1002/cam4.3297

**Published:** 2020-08-04

**Authors:** Kirsti Aas, Sophie Dorothea Fosså, Tor Åge Myklebust, Bjørn Møller, Rune Kvåle, Ljiljana Vlatkovic, Viktor Berge

**Affiliations:** ^1^ Department of Surgery Vestre Viken Hospital Trust Drammen Norway; ^2^ Department of Oncology Oslo University Hospital (OUH) Oslo Norway; ^3^ Cancer Registry of Norway Oslo Norway; ^4^ University of Oslo Oslo Norway; ^5^ Department of Research and Innovation Møre and Romsdal Hospital Trust Alesund Norway; ^6^ Department of Oncology Haukeland University Hospital Bergen Norway; ^7^ Department of Health Registry‐based Research and Development Norwegian Institute of Public Health Bergen Norway; ^8^ Department of Pathology OUH Oslo Norway; ^9^ Department of Urology OUH Oslo Norway

**Keywords:** elderly, mortality, prostate cancer, senior adults, treatment

## Abstract

**Background:**

The association between curative treatment (CurTrt) and mortality in senior adults (≥70 years) with high‐risk prostate cancer (PCa) is poorly documented. In a population‐based cohort we report temporal trends in treatment and PCa‐specific mortality (PCSM), investigating the association between CurTrt and mortality in senior adults with high‐risk PCa, compared to findings in younger men (<70 years).

**Methods:**

Observational study from the Cancer Registry of Norway. Patients with high‐risk PCa were stratified for three diagnostic periods (2005‐08, 2009‐12 and 2013‐16), age (<70, vs ≥70) and primary treatment (CurTrt: Radical prostatectomy (RP), Radiotherapy (RAD) vs no curative treatment (NoCurTrt)). Competing risk and Kaplan‐Meier methods estimated PCSM and overall mortality (OM), respectively. Multivariable logistic regression models estimated odds for CurTrt, and multivariable Fine Gray and Cox regression models evaluated the hazard ratios for PCSM and OM.

**Results:**

Of 19 763 evaluable patients, 54% were aged ≥70 years. Senior adults had more unfavorable PCa characteristics than younger men. Across diagnostic periods, use of CurTrt increased from 15% to 51% in men aged ≥70 and 65% to 81% in men aged < 70 years. With median five years follow‐up, PCSM decreased in all patients (*P* < .05), in the third period restricted to senior adults. In all patients NoCurTrt was associated with three‐fold higher 5‐year PCSM and two‐fold higher OM compared to CurTrt.

**Conclusions:**

In high‐risk PCa patients, increased use of CurTrt, greatest in senior men, was observed along with decreased PCSM and OM in both senior and younger adults. CurTrt should increasingly be considered in men ≥70 years.

## Graphical abstract

Curative treatment in senior adults with high‐risk prostate cancer is associated with decreased prostate cancer‐specific and overall mortality

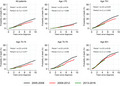

## 1 INTRODUCTION

Prostate cancer (PCa) is a major cause of cancer mortality in senior men worldwide.^1^, ^2^ Due to demographic changes, the number of new PCa cases in men ≥ 70 years is expected to double within year 2040.^3^ According to the literature, a higher proportion of senior adults have high‐risk disease at presentation compared to younger men.^4^, ^5^, ^6^

Elderly patients are underrepresented in clinical trials, and there is no consensus on the optimal treatment strategy in senior adults with high‐risk PCa.^7^, ^8^ The Scandinavian Prostate Cancer Group (SPCG) 4 study demonstrated a survival benefit from radical prostatectomy (RP) compared to watchful waiting in both senior and younger men with localized disease and long life expectancy (LE).^9^ In patients with high‐risk disease, there is level 1 evidence that androgen deprivation therapy (ADT) combined with radiotherapy (RAD) improves survival compared to either modality alone, also in senior adults.^10^, ^11^, ^12^, ^13^ Patients without distant metastases and LE > 5‐10 years, should be considered for curatively intended treatment with RP and extended pelvic lymph node dissection or high‐dose RAD combined with (neo‐) adjuvant ADT.^14^, ^15^, ^16^, ^17^, ^18^ Those who are unwilling or ineligible for curative treatment (CurTrt), may be managed with watchful waiting or ADT.^14^, ^15^, ^16^, ^17^, ^18^

Senior adults with high‐risk PCa comprise a heterogeneous group of patients in terms of PCa characteristics, health status, and LE. Any life‐prolonging effect from CurTrt must in these patients be weighed against the risk of adverse treatment‐related effects and death from other causes than PCa.^19^, ^20^, ^21^ Studies have indicated that treatment decisions in PCa patients are primarily based on chronological age rather than biological age.^7^, ^22^, ^23^, ^24^, ^25^, ^26^, ^27^ Undertreatment of healthy senior adults with high‐risk PCa may thus contribute to the described high incidence of death from PCa in the elderly population.^5^, ^6^, ^20^, ^21^

With this background, we compare patient characteristics, primary treatment, and prostate cancer‐specific mortality (PCSM) in senior adults (≥70 years) and younger men (<70 years) diagnosed with high‐risk PCa in Norway. Furthermore, we investigate the association between CurTrt and mortality in the two age groups.

## 2 METHODS

### 2.1 Data sources

The Norwegian Prostate Cancer Registry is a national clinical quality registry managed by the Cancer Registry of Norway.^28^ The registry codes individual demographic and clinical information, including date of PCa diagnosis, Eastern Cooperative Oncology Group (ECOG) performance status, PSA level, Gleason score, clinical TNM‐categories, and date of RP. The Radiotherapy Database contains information on start of RAD, target site, and target dose from all radiotherapy centers in Norway. Information on the date and cause of death is collected from the Cause of Death Registry. The study was approved by the Regional Committee for Medical and Health Research Ethics (2011/1746).

### 2.2 Patients

Patients diagnosed from 2005 to 2016 with PCa without distant metastases were identified (Appendix 1). For inclusion in the study, the European Association of Urology high‐risk group criteria had to be met, including both localized and locally advanced disease.^29^ Detailed information on clinical N‐category was not available in the registry. Patients were stratified according to diagnostic period (2005‐08, 2009‐12, 2013‐16), age at diagnosis (<70, 70‐74, 75‐79, ≥80 years), and curative treatment (CurTrt: RP, RAD vs no curative treatment (NoCurTrt)). RP was performed in ≤ 12 months of diagnosis. Performance of pelvic lymph node dissection was not reliably documented. RAD was in the current study defined as RAD doses of ≥ 74 Gy, with or without (neo‐) adjuvant ADT, started ≤ 15 months of diagnosis in patients diagnosed 2005‐13 and ≤ 12 months in patients diagnosed 2014‐16. Patients not fulfilling the criteria for primary RP or RAD were allocated to the NoCurTrt group. Treatment was analyzed as a time‐varying covariate. Patients were observed from the time of diagnosis to emigration, death, or end of study date (31st December 2017).

### 2.3 Statistical methods

Standard descriptive methods were applied (frequencies/proportions, medians/ranges). The Chi square tested intergroup differences. Multivariable logistic regression models estimated the odds ratios (ORs) and 95% confidence intervals (CIs) for performance of CurTrt. PCSM was estimated using the Aalen‐Johansen estimator, and mortality estimates were compared using a univariate Fine‐Gray regression model. Overall survival was assessed with the Kaplan‐Meier method. Multivariable Fine‐Gray and Cox regression models tested the relationship (subdistribution hazard ratios (SHRs), hazard ratios (HRs) and CIs) between primary treatment and PCSM and OM, respectively, adjusting for relevant clinical confounding variables available at the time of diagnosis. The level of significance was *P* < .05. Data were analysed using the IBM Statistical Package for the Social Sciences Statistics version 26 and Stata version 14.2.

## 3 RESULTS

### 3.1 Disease characteristics

In total, 19 763 patients with high‐risk PCa were evaluable for the present study (Appendix 1,2). More than half of the patients were aged ≥ 70 years (Table S1). Compared to younger patients, senior adults had poorer ECOG performance status, higher PSA levels, and more unfavorable International Society of Urological pathology (ISUP) grade groups and cT‐categories. Decrease in PSA levels and increase in ISUP grade groups were observed across diagnostic periods, similar for senior and younger patients.

### 3.2 CurTrt vs NoCurTrt

In all patients the use of CurTrt increased from 37% in 2005‐08 to 66% in 2013‐16, with a larger increase in senior adults (≥70 years; 15 to 51%, <70 years: 65 to 81%) (Table 1). Compared to 24% of the younger men, 67% of high‐risk senior adults did not receive CurTrt. Use of RP increased fourfold in patients aged 70‐74 years, and RAD increased sevenfold in patients aged 75‐79 years, whereas RP doubled in the younger ones parallel with decrease in RAD.

**TABLE 1:** Primary treatment in patients diagnosed with high‐risk prostate cancer in Norway

Diagnostic period	2005‐2008					2009‐12					2013‐16					
Age (y)	<70	70‐74	75‐79	≥80	All	<70	70‐74	75‐79	≥80	All	<70	70‐74	75‐79	≥80	All	Total
Patients (n)	2677	1159	1164	1268	6268	3052	1238	1032	1207	6529	3352	1439	1100	1075	6966	19 763
Primary treatment																
RP	824 (31)^a^	72 (6)	11 (<1)	2 (<1)	909 (15)	1484 (49)	231 (19)	20 (2)	2 (<1)	1737 (27)	1972 (59)	433 (30)	66 (6)	0	2471 (36)	5117 (26)
RAD	903 (34)	379 (33)	83 (7)	3 (<1)	1368 (22)	971 (32)	577 (47)	323 (31)	36 (3)	1907 (29)	742 (22)	672 (47)	536 (49)	124 (12)	2074 (30)	5349 (27)
NoCurTrt	950 (36)	708 (61)	1070 (92)	1263 (>99)	3991 (64)	597 (20)	430 (35)	689 (67)	1169 (97)	2885 (44)	638 (19)	334 (23)	498 (45)	951 (89)	2421 (35)	9297 (47)

Abbreviations: NoCurTrt, no curative treatment; post‐RP RAD, post radical prostatectomy radiotherapy; RP, radical prostatectomy.

^a^ Number of patients (% of patients within diagnostic period and age group).

Patients in the NoCurTrt group were older, had poorer ECOG performance status and higher PSA‐levels compared to curatively treated patients (Table S2). In both the CurTrt and NoCurTrt groups, one in two patients had ISUP grade group ≥ 4 tumors.

In multivariable analyses, the odds of receiving CurTrt increased sixfold across the diagnostic periods in senior adults compared to a twofold increase in younger patients (Table 2). For all patients, the probability of receiving CurTrt decreased with increasing age, ECOG status ≥ 1, and a prior cancer diagnosis. In both senior and younger patients, having ISUP grade group ≥ 2 tumors doubled the odds for treatment compared to ISUP grade group 1 tumors.

**TABLE 2:** Logistic regression with curative treatment (RP or RAD) as dependent variable

Age (y)	<70			≥70		
Patients analyzed (n)	7567			8571		
	Odds ratio	95% CI	*P*‐value	Odds ratio	95% CI	*P*‐value
Diagnostic period						
2005‐08	1			1		
2009‐12	2.20	1.92‐2.53	.000	3.44	2.96‐4.00	.000
2013‐16	2.10	1.83‐2.42	.000	6.44	5.49‐7.55	.000
Age						
<60	1					
60‐64	0.89	0.76‐1.04	.140			
65‐69	0.78	0.67‐0.91	.001			
70‐74				1		
75‐79				0.26	0.23‐0.30	.000
≥80				0.03	0.03‐0.04	.000
ECOG						
0	1			1		
1	0.68	0.58‐0.81	.000	0.53	0.46‐0.60	.000
≥2	0.24	0.19‐0.31	.000	0.16	0.13‐0.20	.000
Prior cancer						
No	1			1		
Yes	0.75	0.60‐0.93	.009	0.70	0.58‐0.84	.000
PSA (ng/mL)						
<10	1			1		
10‐20	1.01	0.87‐1.18	.889	0.84	0.72‐0.97	.022
>20	0.44	0.39‐0.50	.000	0.41	0.36‐0.48	.000
ISUP grade group						
1	1			1		
2	2.17	1.81‐2.58	.000	2.26	1.81‐2.83	.000
3	2.05	1.69‐2.48	.000	2.56	2.03‐3.23	.000
4	1.83	1.55‐2.18	.000	2.44	1.97‐3.01	.000
5	1.33	1.10‐1.62	.003	2.26	1.80‐2.84	.000
cT‐category						
1‐2	1			1		
3‐4	0.73	0.65‐0.82	.000	0.89	0.79‐1.01	.066

Abbreviations: CI, confidence interval; cT‐category, clinical tumor‐category; ECOG, Eastern Cooperative Oncology Group functional status; ISUP grade group, International Society of Urological Pathology grade group; PSA, prostate specific antigen; RAD, radiotherapy; RP: radical prostatectomy.

### 3.3 Mortality

**FIGURE 1:**
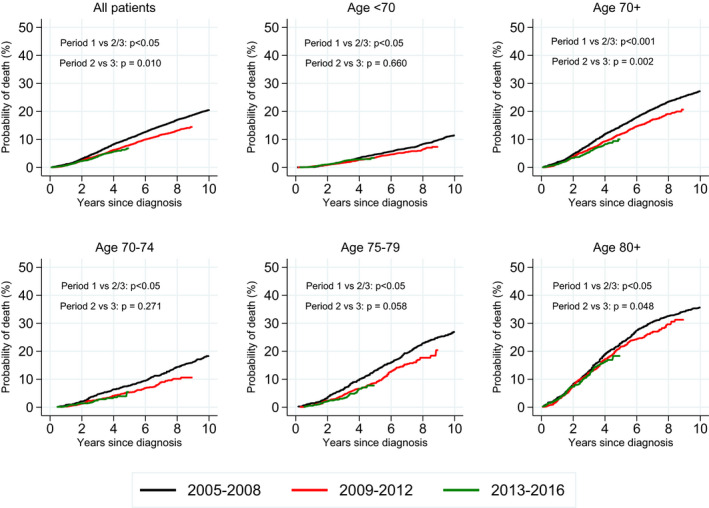
Prostate cancer‐specific mortality according to age group and diagnostic period in patients diagnosed with high‐risk prostate cancer in Norway 2005‐16

**FIGURE 2:**
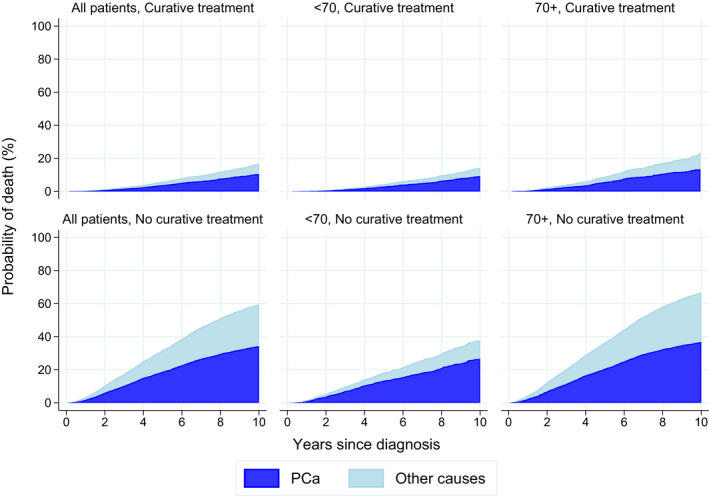
Prostate cancer and other cause mortality according to age group and primary treatment in men diagnosed with high‐risk prostate cancer in Norway 2005‐16. PCa; prostate cancer

For all patients, PCSM decreased within the observation period (*P* < .05), although a decrease in the last diagnostic period was observed only in patients ≥ 70 years (Figure 1). The 5‐year PCSM was 8.8% and OM was 21.8%, both increasing with age at diagnosis (Table 3). With a median follow‐up time of five years (range 0‐13 years), about two of five deaths were caused by PCa in both senior and younger adults (1825/4650 and 570/1307 deaths respectively) (Table 3, Figure 2).

**TABLE 3:** 5‐year prostate cancer‐specific and overall mortality in patients diagnosed with high‐risk prostate cancer

(A)				
Treatment	RP	RAD	NoCurTrt	All
Dead PCa				
All ages	110/5117 (2)^a^	209/5349 (4)	2076/9297 (22)	2395/19763 (12)
<70	90/4280 (2)	123/2616 (5)	357/2185 (16)	570/9081 (6)
70‐74	19/736 (3)	60/1628 (4)	307/1472 (21)	386/3836 (10)
75‐79	1/97 (1)	23/942 (2)	505/2257 (22)	529/3296 (16)
≥80	0/4	3/163 (2)	907/3383 (27)	910/3550 (26)
Dead any cause				
All ages	330/5117 (6)	733/5349 (14)	4894/9297 (53)	5957/19763 (30)
<70	251/4280 (6)	360/2616 (14)	696/2185 (32)	1307/9081 (14)
70‐74	70/736 (10)	236/1628 (14)	691/1472 (47)	997/3836 (26)
75‐79	7/97 (7)	121/942 (13)	1234/2257 (55)	1362/3296 (41)
≥80	2/4 (50)	16/163 (9)	2273/3383 (67)	2291/3550 (65)

(B)				
Treatment	RP	RAD	NoCurTrt	All
PCa‐specific mortality				
All	1.4 (1.0‐1.8)^b^	2.2 (1.8‐2.7)	15.6 (14.9‐16.4)	8.8 (8.4‐9.3)
<70	1.1 (0.8‐1.5)	2.4 (1.8‐3.1)	11.0 (9.6‐12.5)	4.0 (3.6‐4.5)
70‐74	2.8 (1.5‐4.7)	2.0 (1.4‐3.0)	11.3 (9.7‐13.1)	6.1 (5.3‐7.0)
75‐79	2.8 (0.2‐12.4)	2.0 (1.1‐3.5)	13.1 (11.7‐14.7)	10.4 (9.3‐11.6)
≥80	—	3.3 (0.8‐8.9)	22.6 (21.1‐24.1)	22.1 (20.6‐23.6)
Overall mortality				
All	4.7 (4.0‐5.5)	8.4 (7.6‐9.3)	36.2 (35.1‐37.2)	21.8 (21.2‐22.5)
<70	3.8 (3.1‐4.6)	7.0 (6.0‐8.1)	19.9 (18.1‐21.8)	8.9 (8.3‐9.7)
70‐74	10.4 (7.7‐14.0)	8.9 (7.5‐10.7)	26.5 (24.2‐29.0)	16.9 (15.6‐18.3)
75‐79	7.9 (2.8‐21.3)	11.2 (8.9‐14.1)	31.8 (29.8‐33.9)	26.8 (25.2‐28.6)
≥80	25.0 (4.0‐87.2)	20.4 (12.2‐33.0)	55.1 (53.3‐57.0)	54.2 (52.4‐56.0)

Abbreviations: NoCurTrt: no curative treatment; PCa: prostate cancer; RAD: radiotherapy; RP: radical prostatectomy.

^a^ Mortality rate % (95% confidence interval).

^b^ Number of patients (% within treatment group).

In multivariable analyses, NoCurTrt was associated with more than threefold increased risk of PCa death in all patients, with similar results for RP and RAD (Table 4, Figure S1). Time‐dependent decreasing probabilities of PCa death with increasing diagnostic periods were further reduced if primary treatment was excluded from the analysis (data not shown). Having ISUP grade group five vs one increased the HR of death from PCa almost four times (95% CI 2.9‐4.3) in men ≥ 70 years compared to 13 times (95% CI 8.5‐22.1) in men < 70 years (Table 4). In both senior and younger adults, NoCurTrt more than doubled the overall 5‐year risk of death (Table 5). The HR of OM decreased with increasing diagnostic period in senior adults (Table 5), also when defining death from PCa as a competing risk (data not shown).

**TABLE 4:** Multivariable Fine‐Gray regression with prostate cancer‐specific mortality as dependent variable in patients diagnosed with high‐risk prostate cancer

Age (years)	<70						≥70					
Patients analyzed (n)	7567						8563					
Analysis	Univariable			Multivariable			Univariable			Multivariable		
	SHR	95% CI	*P*‐value	SHR	95% CI	*P*‐value	SHR	95% CI	*P*‐value	SHR	95% CI	*P*‐value
Diagnostic period												
2005‐08	1			1			1			1		
2009‐12	0.74	0.61‐0.89	.002	0.72	0.58‐0.91	.006	0.69	0.63‐0.77	.000	0.70		.000
2013‐16	0.79	0.59‐1.04	.097	0.64	0.42‐0.92	.017	0.53	0.46‐0.62	.000	0.48		.000
Treatment												
RP	1			1			1			1		
RAD	1.71	1.30‐2.24	.000	1.02	0.75‐1.40	.893	1.12	0.69‐1.81	.648	0.90	0.52‐1.53	.685
NoCurTrt	6.02	4.76‐7.62	.000	3.16	2.36‐4.23	.000	6.93	4.47‐10.74	.000	3.69	2.24‐6.09	.000
Age												
<60	1			1								
60‐64	1.10	0.87‐1.38	.419	0.91	0.70‐1.19	.498						
65‐69	1.17	0.94‐1.45	.158	0.91	0.71‐1.18	.490						
70‐74							1			1		
75‐79							1.59	1.40‐1.81	.000	1.08	0.94‐1.25	.283
≥80							2.75	2.44‐3.09	.000	1.35	1.17‐1.56	.000
ECOG												
0	1			1			1			1		
1	2.03	1.62‐2.55	.000	1.43	1.11‐1.83	.006	1.46	1.30‐1.64	.000	1.11	0.98‐1.26	.100
≥2	1.54	1.03‐2.30	.034	0.79	0.50‐1.24	.303	1.80	1.59‐2.04	.000	1.07	0.93‐1.23	.357
PSA (ng/mL)												
<10	1			1			1			1		
10‐20	1.47	1.14‐1.89	.003	1.11	0.84‐1.46	.474	1.29	1.09‐1.53	.003	1.06	0.89‐1.26	.535
>20	2.27	1.83‐2.82	.000	1.50	1.17‐1.92	.002	1.86	1.61‐2.15	.000	1.27	1.08‐1.48	.003
ISUP grade group												
1	1			1			1			1		
2	2.46	1.54‐3.93	.000	2.57	1.59‐4.15	.000	1.18	0.95‐1.47	.131	1.24	0.99‐1.55	.067
3	4.20	2.64‐6.69	.000	3.67	2.25‐5.98	.000	1.59	1.28‐1.97	.000	1.63	1.29‐2.05	.000
4	5.55	3.59‐8.59	.000	5.73	3.65‐8.99	.000	1.96	1.61‐2.38	.000	2.15	1.75‐2.65	.000
5	16.72	10.89‐25.67	.000	13.38	8.49‐22.08	.000	3.54	2.93‐4.29	.000	3.61	2.93‐4.46	.000
cT‐category												
1‐2	1			1			1			1		
3‐4	2.05	1.70‐2.46	.000	1.45	1.18‐1.78	.000	1.45	1.31‐1.60	.000	1.30	1.17‐1.45	.000

Abbreviations: CI, confidence interval; cT‐category, clinical tumor‐category; ECOG, Eastern Cooperative Oncology Group functional status; ISUP grade group, International Society of Urological Pathology grade group; NoCurTrt, no curative treatment; PSA, prostate specific antigen; RAD, radiotherapy, SHR, sub‐distribution hazard ratio; RP, radical prostatectomy.

**TABLE 5:** Cox regression with overall mortality as dependent variable in patients diagnosed with high‐risk prostate cancer

Age (y)	<70			≥70		
Patients analyzed (n)	7567			8563		
	HR	95% CI	*P*‐value	HR	95% CI	*P*‐value
Diagnostic period						
2005‐08	1			1		
2009‐12	0.95	0.81‐1.10	.458	0.86	0.80‐0.93	.000
2013‐16	1.00	0.79‐1.27	.991	0.81	0.72‐0.92	.001
Treatment						
RP	1			1		
RAD	1.29	1.07‐1.55	.007	0.96	0.73‐1.26	.762
NoCurTrt	2.74	2.29‐3.28	.000	2.25	1.73‐2.93	.000
Age						
<60	1					
60‐64	1.15	0.96‐1.39	.133			
65‐69	1.50	1.27‐1.78	.000			
70‐74				1		
75‐79				1.30	1.18‐1.43	.000
≥80				2.14	1.95‐2.35	.000
ECOG						
0	1			1		
1	1.81	1.55‐2.11	.000	1.31	1.21‐1.41	.000
≥2	2.34	1.91‐2.87	.000	1.79	1.64‐1.94	.000
PSA (ng/mL)						
<10	1			1		
10‐20	1.31	1.10‐1.54	.002	1.14	1.02‐1.27	.019
>20	1.52	1.30‐1.78	.000	1.34	1.22‐1.47	.000
ISUP grade group						
1	1			1		
2	1.30	1.06‐1.61	.013	1.07	0.94‐1.21	.296
3	1.51	1.21‐1.88	.000	1.27	1.12‐1.43	.000
4	1.73	1.41‐1.12	.000	1.48	1.32‐1.66	.000
5	2.95	2.39‐3.65	.000	1.94	1.73‐2.19	.000
cT‐category						
1‐2	1			1		
3‐4	1.15	1.01‐1.30	0.035	1.18	1.10‐1.26	0.000

Note

Abbreviations: CI: confidence interval; cT‐category: clinical tumor‐category; ECOG: Eastern Cooperative Oncology Group functional status; HR: Hazard ratio; ISUP grade group: International Society of Urological Pathology grade group; NoCurTrt: no curative treatment; PSA: prostate specific antigen; RAD: radiotherapyRP: radical prostatectomy.

## 4 DISCUSSION

In this population‐based cohort of high‐risk PCa patients, increased use of CurTrt, greatest in senior men, was observed with time, along with decreased PCSM and OM in both senior and younger adults.

### 4.1 Disease characteristics

Our findings are in agreement with previous studies demonstrating higher prevalence of high‐risk disease in senior adults compared to younger men.^4^, ^5^ Admittedly, more aggressive histology observed with time in all patients, may relate to gradual implementation of the 2005 ISUP Gleason score modifications and increased use of targeted biopsies.

### 4.2 Treatment

In our study, we observed a striking increase in the use of RP in patients up to 75 years of age and RAD in senior adults. Improvements in CurTrt techniques with lower toxicity, along with increasing LE in the population, may have influenced physicians´ decisions. Furthermore, increased use of CurTrt may relate to a 2012 consensus, stating that CurTrt should be discussed with high‐risk patients having LE more than 5 years.^18^

Recommendations advocating management of senior adults according to health status rather than chronological age, were not implemented in the European Association of Urology Guidelines until 2016, but preceding discussions within the uro‐oncological community may have guided clinical practice earlier.^30^ Furthermore, the overall increase in use of CurTrt across diagnostic periods reflects the increase in ISUP grade groups, being a strong predictor of unfavorable outcomes.^31^

### 4.3 Mortality

The decrease in PCSM coincides with the increase in use of CurTrt during the observation period, without differences for RP and RAD. Adjusted for well‐known prognostic risk factors, CurTrt was associated with reduced likelihood of 5‐year PCSM and OM in both senior and younger adults. In contrast with our findings, the SPCG‐4 trial did not show a survival benefit until more than 20 years follow‐up with RP compared to watchful waiting in patients aged 65‐75 years, however, only 3% had Gleason score 8‐10 tumors.^9^, ^32^ Our results are in agreements with retrospective series, demonstrating reduced 5‐10‐year PCSM with local treatment in patients aged ≥ 75 years with ISUP grade group ≥ 2 and locally advanced tumors.^33^, ^34^

In line with our findings regarding PCSM, relative survival is reduced in Norwegian PCa patients aged > 70 years at diagnosis, with a marked reduction in patients ≥ 80 years.^35^ Similarly, a previous population‐based study demonstrated reduced 10‐year relative survival in senior adults compared with younger men, with more pronounced differences in high‐risk patients.^6^ Unlike previously reported, ECOG status was no longer an independent predictor of PCSM in senior adults when analyzed in a competing risk setting (Table 4, Table S3).^36^ Surprisingly, increasing ISUP grade groups were associated with higher risk of PCa death in younger than in senior men (Table 4). We speculate whether this finding may be related to underlying host‐related differences, such as reduced free testosterone levels, associated with age.^37^, ^38^, ^39^

### 4.4 Undertreatment of senior adults

Even in the most recent period in this study, almost half of the senior adults with high‐risk PCa did not receive CurTrt. Comparable to findings by Rider et al and Albertsen et al, a considerable proportion of these patients died from PCa.^20^, ^21^ Similar to younger men, senior adults in the NoCurTrt group had a twofold increased risk of overall death within five years of diagnosis compared to patients treated curatively. The considerable 5‐year PCSM rates, along with the high proportion of patients having ISUP grade group ≥ 4 tumors and ECOG status ≤ 1 in the NoCurTrt group, suggest the likelihood of undertreatment, as also emphasized in other studies.^26^, ^27^

### 4.5 Treatment decisions in senior adults

Higher age is associated with more peri‐operative complications and poorer functional outcomes after radical treatment,^4^, ^19^, ^41^, ^42^ although, several studies report tolerable side‐effects with CurTrt in senior adults.^43^, ^44^ The possibility of undertreatment and early death from high‐risk PCa may imply that the selection criteria for CurTrt are too strict and LE may be underestimated. When CurTrt is considered in senior adults, formal health assessment and individual in‐depth patient counseling are obligatory to facilitate optimal patient selection.

### 4.6 Limitations and strengths

This registry‐based cohort study has several limitations. Our cohort presents a minimum estimate since data were insufficient for risk grouping in 8347 of the initial 26 819 patients without distant metastases (31%). A case mix, with stage and grade migration, may have occurred during the study period, resulting from improvements in diagnostic methods. Major limitations include the lack of detailed comorbidity data. Furthermore, complete data on ADT use, disease progression and second‐line cancer treatments were not available in the Norwegian Prostate Cancer Registry. Estimation of PCSM was based on official cause of death registration and over‐/underreporting of PCa as cause of death, particularly in senior adults, must be considered.^45^, ^46^, ^47^, ^48^ The strengths of this study include real‐life data from a large population‐based cohort of senior adults with high‐risk PCa, assessing the association between CurTrt and PCSM.

## 5 CONCLUSION

Use of CurTrt increased with time and was associated with decreased PCSM and OM in senior adults with high‐risk PCa, suggesting that CurTrt may benefit appropriately selected patients.

## 6 CREDIT AUTHOR STATEMENT

**Aas:** Conceptualization, methodology, formal analysis, investigation, writing – original draft, writing – review and editing, visualization, project administration, funding acquisition. **Fosså**: Conceptualization, methodology, formal analysis, investigation, writing – original draft, writing – review and editing, visualization, supervision, project administration, funding acquisition. **Myklebust:** Methodology, formal analysis, investigation, writing – review and editing, visualization. **Møller:** Conceptualization, methodology, investigation, writing – review and editing. **Kvåle**: writing – review and editing. **Vlatkovic:** writing – review and editing. **Berge:** Conceptualization, methodology, writing – original draft, writing – review and editing, visualization, supervision, project administration.

## CONFLICTS OF INTEREST

None of the authors have conflicts of interests to disclose.

## AUTHORS CONTRIBUTION

All authors listed on the title page have made substantial contributions to the manuscript and agree to the submission to Cancer Medicine.

## Supporting information

Fig S1

Table S1

Table S2

Table S3

Supplementary Material

Supplementary Material

## Data Availability

The data that support the findings of this study are available from the Cancer Registry of Norway Restrictions apply to the availability of these data, which were used under license for this study. Data are available from the authors with the permission of the Cancer Registry of Norway.
