# Supplementary material for: Increased curative treatment is associated with decreased prostate cancer‐specific and overall mortality in senior adults with high‐risk prostate cancer; results from a national registry‐based cohort study
Source: Cancer Med. 2020 Aug 4;9(18):6646–57. doi: 10.1002/cam4.3297 (PMC7520350; doi:10.1002/cam4.3297)
Supplement: Supplementary file 2 — Table S1 [file CAM4-9-6646-s002.docx]

**Supplementary Table 1: Characteristics of patients diagnosed with high-risk prostate cancer in Norway 2005-16**

| Diagnosis period | 2005-08 | | | 2009-12 | | | 2013-16 | | | All periods | | |
| --- | --- | --- | --- | --- | --- | --- | --- | --- | --- | --- | --- | --- |
| Age group (years) | **<70** | **≥70** | **All** | **<70** | **≥70** | **All** | **<70** | **≥70** | **All** | **<70** | **≥70** | **All** |
| Number of patients | 2677 (43) | 3591 (57) | 6268 | 3052 (47) | 3477 (53) | 6529 | 3352 (48) | 3614 (52) | 6966 | 9081 (46) | 10682 (54) | 19763 |
| ECOG  0  1  ≥2  Missing | 2095 (78)  284 (11)  104(4)  194 (7) | 1561 (44)  1000 (28)  774 (22))  256 (7) | 3656 (58)  1284 (21)  878 (14)  450 (7) | 2145 (70)  317 (10)  114 (4)  476 (16) | 1436 (41)  900 (26)  638 (18))  503 (15) | 3581 (55)  1217 (19)  752 (12)  979 (15) | 2313 (69)  283 (8)  82 (2)  674 (20) | 1620 (45)  727 (20)  410 (11)  857 (24) | 3933 (57)  1010 (15)  492 (7)  1531 (22) | 6553 (72)  884 (10)  300 (3)  1344 (15) | 4617 (43)  2627 (25)  1822 (17)  1616 (15) | 11170 (57)  3511 (18)  2122 (11)  2960 (15) |
| Prior cancer  No  Yes | 2519 (94)  158 (6) | 3169 (88)  422 (12) | 5688 (91)  580 (9) | 2835 (93)  217 (7) | 3019 (87)  458 (13) | 5854 (90)  675 (10) | 3137 (94)  215 (6) | 3085 (85)  529 (15) | 6222 (89)  744 (11) | 8491 (94)  590 (7) | 9273 (87)  1409 (13) | 17764 (90)  1999 (10) |
| PSA (ng/mL)  <10  10-20  >20  Missing | 845 (32)  671 (25)  1053 (39)  108 (4) | 527 (15)  790 (22)  2131 (59)  143 (4) | 1372 (22)  1461 (23)  3184 (51)  251 (4) | 1142 (37)  729 (24)  936 (31)  245 (8) | 681 (20)  878 (25)  1619 (47)  299 (9) | 1823 (28)  1607 (25)  2555 (39)  544 (8) | 1634 (49)  656 (20)  700 (21)  362 (11) | 1015 (28)  888 (25)  1122 (31)  589 (16) | 2649 (38)  1544 (22)  1822 (26)  951 (14) | 3621 (40)  2056 (23)  2689 (30)  715 (8) | 2223 (21)  2556 (24)  4872 (46)  1031 (10) | 5844 (30)  4612 (23)  7561 (38)  1746 (9) |
| ISUP grade group  1  2  3  4  5  Missing | 616 (23)  566 (21)  695 (26)  409 (15)  579 (22)  270 (10) | 546 (15)  803 (22)  551 (15)  918 (26)  614 (17)  159 (4) | 1162 (19)  1498 (24)  960 (15)  1497 (34)  884 (14)  267 (4) | 371 (12)  682 (22)  498 (16)  963 (32)  496 (16)  42 (1) | 334 (10)  539 (16)  541 (16)  1185 (34)  802 (23)  76 (2) | 705 (11)  1221 (19)  1039 (16)  2148 (33)  1298 (20)  118 (2) | 331 (10)  741 (22)  516 (15)  1053 (31)  686 (21)  25 (<1) | 218 (6)  487 (14)  530 (15)  1249 (35)  1078 (30)  52 (1) | 549 (8)  128 (18)  1046 (15)  2302 (33)  1764 (25)  77 (1) | 1318 (15)  2118 (23)  1423 (16)  2595 (29)  1452 (16)  175 (2) | 1098 (10)  1829 (17)  1622 (15)  3352 (31)  2494 (23)  287 (3) | 2416 (12)  3947 (20)  3045 (15)  5947 (30)  3946 (20)  462 (2) |
| cT-category  1-2  3-4  Missing | 1190 (45)  1419 (53)  68 (3) | 1440 (40)  1934 (54)  217 (6) | 2630 (42)  3353 (54)  285 (5) | 1644 (54)  1174 (39)  234 (8) | 1675 (48)  1493 (43)  309 (9) | 3319 (51)  2667 (41)  543 (8) | 1691 (50)  1293 (39)  368 (11) | 1477 (41)  1542 (43)  595 (17) | 3168 (46)  2835 (41)  963 (14) | 4525 (50)  3886 (43)  670 (7) | 4592 (43)  4969 (47)  1121 (11) | 9117 (46)  8855 (45)  1791 (9) |
|  |  |  |  |  |  |  |  |  |  |  |  |  |

Abbreviations: ECOG: Eastern Cooperative Oncology Group functional status; PSA: prostate specific antigen; ISUP grade group: International Society of Urological Pathology grade group; cT-category: clinical tumor-category
