# Supplementary material for: Increased curative treatment is associated with decreased prostate cancer‐specific and overall mortality in senior adults with high‐risk prostate cancer; results from a national registry‐based cohort study
Source: Cancer Med. 2020 Aug 4;9(18):6646–57. doi: 10.1002/cam4.3297 (PMC7520350; doi:10.1002/cam4.3297)
Supplement: Supplementary file 3 — Table S2 [file CAM4-9-6646-s003.docx]

**Supplementary Table 2: Characteristics of patients with high-risk disease according to primary treatment and diagnostic period**

|  | CurTrt | | | NoCurTrt | | |
| --- | --- | --- | --- | --- | --- | --- |
| Diagnostic period | **2005-08** | **2009-12** | **2013-16** | **2005-08** | **2009-12** | **2013-16** |
| Number of patients | 2277 | 3644 | 4545 | 3991 | 2885 | 2421 |
| Age (years)  Median (range)  <70  >70 | 65 (42-84)  1727 (76)^[[1]](#footnote-1)^  550 (24) | 67 (45-86)  2455 (67)  1189 (33) | 68 (40-87)  2714 (60)  1831 (40) | 76 (44-100)  950 (24)  3041 (76) | 78 (46-98)  597 (21)  2288 (79) | 77 (40-96)  638 (26)  1783 (74) |
| ECOG  0  1  ≥2  Missing | 1799 (79)  256 (11)  61 (3)  161 (7) | 2530 (69)  474 (13)  94 (3)  546 (15) | 2997 (66)  530 (12)  118 (3)  900 (20) | 1857 (47)  1028 (26)  817 (21)  289 (7) | 1051 (36)  743 (26)  658 (23)  433 (15) | 936 (39)  480 (20)  374 (15)  631 (26) |
| Prior cancer  Yes  No | 130 (6)  2147 (94) | 262 (7)  3382 (93) | 383 (8)  4162 (92) | 450 (11)  3541 (89) | 413 (14)  2472 (86) | 361 (15)  2060 (85) |
| PSA (ng/mL)  Median (range)  <10  10-20  >20  Missing | 13 (0-100)  809 (36)  676 (30)  702 (31)  90 (4) | 13 (1-100)  1342 (37)  967 (27)  1059 (29)  276 (8) | 10 (1-100)  2021 (45)  1093 (24)  944 (21)  487 (11) | 26 (0-100)  563 (14)  785 (20)  2482 (62)  161 (4) | 23 (0-100)  481 (17)  640 (22)  1496 (52)  268 (9) | 17 (1-100)  628 (26)  451 (19)  878 (36)  464 (19) |
| ISUP grade group  1  2  3  4-5  Missing | 504 (22)  636 (28)  340 (15)  711 (31)  86 (4) | 342 (9)  817 (22)  612 (17)  1841 (51)  32 (1) | 245 (5)  889 (20)  750 (17)  2636 (58)  25 (1) | 658 (17)  862 (22)  620 (16)  1670 (42)  181 (5) | 363 (13)  404 (14)  427 (15)  1605 (56)  86 (3) | 304 (13)  339 (14)  296 (12)  1430 (59)  52 (2) |
| cT-category  1-2  3-4  Missing | 1065 (47)  1171 (51)  41 (2) | 1988 (55)  1391 (38)  265 (7) | 2154 (47)  1892 (42)  499 (11) | 1565 (39)  2182 (55)  244 (6) | 1331 (46)  1276 (44)  278 (10) | 1014 (42)  943 (39)  464 (19) |

Abbreviations: CurTrt: curative treatment; NoCurTrt: no curative treatment; ECOG: Eastern Cooperative Oncology Group functional status; PSA: prostate specific antigen; ISUP grade group: International Society of Urological Pathology grade group

1. Number of patients (% within treatment group and diagnostic period) [↑](#footnote-ref-1)
