# Supplementary material for: Increased curative treatment is associated with decreased prostate cancer‐specific and overall mortality in senior adults with high‐risk prostate cancer; results from a national registry‐based cohort study
Source: Cancer Med. 2020 Aug 4;9(18):6646–57. doi: 10.1002/cam4.3297 (PMC7520350; doi:10.1002/cam4.3297)
Supplement: Supplementary file 4 — Table S3 [file CAM4-9-6646-s004.docx]

**Supplementary Table 3: Multivariable Cox regression with prostate cancer-specific mortality as dependent variable in patients with high-risk prostate cancer**

| Age (years) | <70 | | | ≥70 | | |
| --- | --- | --- | --- | --- | --- | --- |
| Patients analyzed (n) | 7567 | | | 8563 | | |
|  | **Hazard ratio** | **95% CI** | **p-value** | **Hazard ratio** | **95% CI** | **p-value** |
| Diagnostic period  2005-08  2009-12  2013-16 | 1  0.80  0.75 | 0.63-1.01  0.50-1.12 | 0.063  0.157 | 1  0.78  0.66 | 0.69-0.88  0.54-0.82 | 0.000  0.000 |
| Treatment  RP  RAD  NoCurTrt | 1  1.03  3.41 | 0.76-1.41  2.57-4.52 | 0.838  0.000 | 1  0.82  3.71 | 0.48-1.41  2.23-6.17 | 0.467  0.000 |
| Age  <60  60-64  65-69  70-74  75-79  ≥80 | 1  0.94  0.97 | 0.73-1.22  0.76-1.25 | 0.657  0.838 | 1  1.16  1.81 | 1.00-1.35  1.56-2.10 | 0.047  0.000 |
| ECOG  0  1  ≥2 | 1  1.59  1.00 | 1.24-2.03  0.66-1.51 | 0.000  0.998 | 1  1.20  1.41 | 1.06-1.36  1.23-1.62 | 0.004  0.000 |
| PSA (ng/mL)  <10  10-20  >20 | 1  1.14  1.55 | 0.87-1.50  1.21-1.97 | 0.332  0.000 | 1  1.09  1.37 | 0.91-1.30  1.17-1.60 | 0.356  0.000 |
| ISUP grade group  1  2  3  4  5 | 1  2.60  3.76  5.83  14.01 | 1.60-4.21  2.31-6.13  3.70-9.17  8.90-22.04 | 0.000  0.000  0.000  0.000 | 1  1.24  1.72  2.34  4.13 | 0.98-1.56  1.37-2.17  1.89-2.88  3.34-5.10 | 0.070  0.000  0.000  0.000 |
| cT-category  1-2  3-4 | 1  1.43 | 1.17-1.75 | 0.000 | 1  1.36 | 1.22-1.51 | 0.000 |

Abbreviations: CI: confidence interval; ECOG: Eastern Cooperative Oncology Group functional status; PSA: prostate specific antigen; ISUP grade group: International Society of Urological Pathology grade group; cT-category: clinical tumor-category; RP: radical prostatectomy; RAD: radiotherapy; NoCurTrt: no curative treatment
