# Supplementary material for: Increased curative treatment is associated with decreased prostate cancer‐specific and overall mortality in senior adults with high‐risk prostate cancer; results from a national registry‐based cohort study
Source: Cancer Med. 2020 Aug 4;9(18):6646–57. doi: 10.1002/cam4.3297 (PMC7520350; doi:10.1002/cam4.3297)
Supplement: Supplementary file 5 — Supplementary Material [file CAM4-9-6646-s005.docx]

**Appendix 1:** Patient cohort

55 316

Diagnosis based on cystoprostatectomy

54 695

Diagnosed incidentally by autopsy or no cytology/histology

51 707

Morphology other than adenocarcinoma

51 596

**Patients diagnosed with prostate cancer (PCa) recorded by the Norwegian Prostate Cancer Registry 2005-2016**

Distant metastasis

47 791

PSA >100 ng/mL

**19 763**

No high-risk features (missing PSA, clinical T-category (cT) and/or Gleason score or cT2x; 8347)

46 582

Radiotherapy to the prostate prior to PCa diagnosis

Duplicate patients

46 583

46 586
