# Supplementary material for: Increased curative treatment is associated with decreased prostate cancer‐specific and overall mortality in senior adults with high‐risk prostate cancer; results from a national registry‐based cohort study
Source: Cancer Med. 2020 Aug 4;9(18):6646–57. doi: 10.1002/cam4.3297 (PMC7520350; doi:10.1002/cam4.3297)
Supplement: Supplementary file 6 — Supplementary Material [file CAM4-9-6646-s006.docx]

**Appendix 2: Patients with high-risk disease among all men diagnosed with prostate cancer without distant metastasis in Norway 2005-16**

| Diagnostic period | 2005-08 | | 2009-12 | | 2013-16 | | All periods | |
| --- | --- | --- | --- | --- | --- | --- | --- | --- |
| Risk group | **High-risk** | **All** | **High-risk** | **All** | **High-risk** | **All** | **High-risk** | **All** |
|  | 6268 (47)^[[1]](#footnote-1)^ | 13341 | 6529 (42) | 15718 | 6966 (40) | 17523 | 19763 (42) | 46582 |
| Age group |  |  |  |  |  |  |  |  |
| <70 | 2677 (37)^[[2]](#footnote-2)^ | 7149 | 3052 (33) | 9310 | 3352 (33) | 10168 | 9081 (34) | 26627 |
| 70-74 | 1159 (49) | 2348 | 1238 (45) | 2772 | 1439 (41) | 3478 | 3836 (45) | 8598 |
| 75-79 | 1164 (59) | 1959 | 1032(55) | 1866 | 1100 (50) | 2185 | 3296 (55) | 6010 |
| ≥80 | 1268 (67) | 1885 | 1207 (68) | 1770 | 1075 (64) | 1692 | 3550 (66) | 5347 |

1. Number of patients (% within diagnostic period) [↑](#footnote-ref-1)
2. Number of patients (% within diagnostic period and age group) [↑](#footnote-ref-2)
